# The effect of depression symptoms and social support on black-white differences in health-related quality of life in early pregnancy: the health status in pregnancy (HIP) study

**DOI:** 10.1186/1471-2393-13-125

**Published:** 2013-06-03

**Authors:** Li Liu, Rosanna Setse, Ruby Grogan, Neil R Powe, Wanda K Nicholson

**Affiliations:** 1 Department of International Health, Johns Hopkins University, Baltimore, USA; 2 Department of Epidemiology, Johns Hopkins University, Baltimore, USA; 3 Department of Medicine University of California, San Francisco, USA; 4 Department of Obstetrics and Gynecology, University of North Carolina, Chapel Hill, USA; 5 Center for Women’s Health Research, University of North Carolina, Chapel Hill, USA; 6 Diabetes Center, University of North Carolina, Chapel Hill, USA

**Keywords:** Depressive symptoms, Race, Pregnancy and health-related quality of life

## Abstract

**Background:**

Lower physical and social functioning in pregnancy has been linked to an increased risk of preterm delivery and low birth weight infants, butt few studies have examined racial differences in pregnant women’s perception of their functioning. Even fewer studies have elucidated the demographic and clinical factors contributing to racial differences in functioning. Our objective was to determine whether there are racial differences in health-related quality of life (HRQoL) in early pregnancy; and if so, to identify the contributions of socio-demographic characteristics, depression symptoms, social support and clinical factors to these differences.

**Methods:**

Cross-sectional study of 175 women in early pregnancy attending prenatal clinics in urban setting. In multivariate analysis, we assessed the independent relation of black race (compared to white) to HRQoL scores from the eight domains of the Medical Outcomes (SF-36) Survey: Physical Functioning, Role-Physical, Bodily Pain, Vitality, General Health, Social Functioning, Role-Emotional, and Mental Health. We compared socio-demographic and clinical factors and depression symptoms between black and white women and assessed the relative importance of these factors in explaining racial differences in physical and social functioning.

**Results:**

Black women comprised 59% of the sample; white women comprised 41%. Before adjustment, black women had scores that were 14 points lower in Physical Function and Bodily Pain, 8 points lower in General Health, 4 points lower in Vitality and 7 points lower in Social Functioning. After adjustment for depression symptoms, social support and clinical factors, black women still had HRQoL scores that were 4 to 10 points lower than white women, but the differences were no longer statistically significant. Level of social support and payment source accounted for most of the variation in Physical Functioning, Bodily Pain and General Health. Social support accounted for most of the differences in Vitality and Social Functioning.

**Conclusions:**

Payment source and social support accounted for much of the racial differences in physical and social function scores. Efforts to reduce racial differences might focus on improving social support networks and Socio-economic barriers.

## Background

Several investigations show that depressive symptoms and social support are important determinants of health-related quality of life (HRQoL) in pregnant women [1-7]. The generalizability of these investigations, however, has been limited since the majority of studies, both in the United States and worldwide, have been conducted in predominately white and middle-class women. Emmanual and colleagues [6], for example, reported that social support was a significant and consistent predictor of HRQoL during the perinatal period, but this study was limited to a sample of predominately white women. Fewer studies have examined differences in HRQoL in a racially and socioeconomically diverse sample of pregnant women [5,8,9]. Even fewer studies have evaluated the individual and combined influence of explanatory factors (demographic, psychosocial or clinical) on physical, emotional and social functioning during the perinatal period or early pregnancy [5,10-12]. Darcy and colleagues [11] reported several factors, including age, black race and marital status, can contribute to postpartum depressive symptoms and poorer HRQoL. Negron and colleagues [12] found that physical symptom burden and social support were important factors in determining depression symptoms and HRQoL among pregnant minority women.

It is important for prenatal clinicians to be knowledgeable of the factors associated with depression symptoms and limited social support among pregnant women. Lower physical functioning prior to conception [13] and in early pregnancy [14] has been linked to an increased risk of preterm birth. Poor emotional functioning has been associated with an increase in prenatal visits, fetal surveillance and resource use [10]. Offspring of women with poorer functioning have worse access and receipt of health care services [15,16].

The purpose of this study was to determine whether black-white differences in maternal perceptions of HRQoL exist, and if so, to elucidate the effects of depressive symptom level, social support and clinical factors on these differences. We hypothesized that black-white differences in HRQoL would exist in early pregnancy and that depressive symptoms would account for much of these differences. Our objectives were to 1) estimate the magnitude of racial differences in perceived HRQoL in early pregnancy and 2) measure the presence, direction, strength, and independence of explanatory factors on HRQoL. If demographic or socioeconomic factors account for differences between black and white women, the development of targeted social interventions would be indicated. Alternatively, if variations in depressive symptoms account for much of the difference in HRQoL, clinicians might develop strategies to reduce the burden depressive symptoms during the preconception and prenatal period.

## Methods

### Study setting and participant eligibility

The study population consisted of pregnant women participating in the Health Status in Pregnancy (HIP) Study, a longitudinal study of functional status during pregnancy and after delivery among a diverse sample of pregnant women in Baltimore city. This study represents an analysis of the baseline data. Women were recruited at the time of the first prenatal visit (termed “new to nurse” visit) at two outpatient clinics in Baltimore city. Women were eligible for enrollment if they were 1) 18 years of age or older, 2) presented for antenatal care at one of two outpatient settings; 3) intended to maintain their pregnancies and deliver within Baltimore city, 4) 14 weeks gestation or less at the time of enrollment and 5) able to provide written consent in English. Women were excluded if they had a diagnosis of human immunodeficiency virus (HIV) or cancer. The study was approved by the institutional review board.

Recruitment was conducted over a 10-month period between July 24, 2004, and May 31, 2005 with final follow-up in May, 2006, at two university-based outpatient clinics. One clinic was located on the university campus. A second clinic was located in the surrounding community within 2 miles of the university hospital. These clinics provide prenatal care to a racially diverse population and include women with Medicaid and commercial insurance. Written informed consent was obtained from each participant by a trained interviewer.

Gestational age at recruitment was based on the last menstrual period (LMP), first trimester ultrasound assessment if it had already been obtained, or both. Gestational age was later confirmed through a review of the electronic medical record and was based on either the obstetrician’s assessment of the LMP or both the LMP and obstetric ultrasound assessment. If there was a discrepancy between the gestational age by LMP and ultrasound, then the gestational age determined by ultrasound was assigned to the participant.

### Dependent variable

HRQoL was measured using the Medical Outcomes Survey Short Form (SF-36), [17] a multidimensional measure of health status designed for self or interviewer administration. The SF-36 has been validated in pregnant women and has been shown to be a reliable tool [18]. There is internal consistency for the SF-36 in the general population (Cronbach’s alpha >0.8), disadvantaged subgroups (> 0.70), [19] and postpartum women (> 0.70). The questionnaire measures perceptions of Physical Functioning, Role-Physical, Bodily Pain, General Health, Vitality, Role-Emotional, Social Functioning and Mental Health. Physical Functioning measures the extent to which health interferes with a variety of physical activities. Role-Physical measures problems with work or other daily activities as a result of physical health. Bodily Pain assesses the extent of bodily pain and related limitations. General Health is a personal evaluation of general health. Vitality provides the perception of degree of fatigue or energy. Social Functioning measures the extent to which health interferes with normal social activities. Role-Emotional reflects problems with work or other activities as a result of emotional problems. Mental Health reflects general mood, psychological well-being, or distress. 1) Responses to questions are scored on a 5-point scale. These absolute scores are then transformed into a score between 0 and 100, with higher scores indicating better functioning or well-being. A score of 100 represents optimal health.

### Independent variable

Maternal race, was based on maternal self-report at the time of presentation for prenatal care. Study participants were classified as: 1) non-Hispanic whites, 2) non-Hispanic blacks, 3) Asian/Pacific Islanders, 4) American Indians, and 5) “other.”

### Covariates

#### Demographics

Socio-demographic variables were abstracted from electronic patient records at baseline and included maternal age, marital status and parity. Socioeconomic factors included employment status (employed, unemployed) at time of study, years of education (less than 12 years, 12 years or more) and insurance (Medicaid, commercial).

#### Depressive symptoms

Depressive symptoms were measured using the Center for Epidemiologic Studies Depression (CES-D) Scale. The Center for Epidemiologic Studies Depression Scale is a 20-item self-report instrument developed by the National Institute of Mental Health to assess depressive symptoms among samples drawn from communities [20]. The reliability and validity of the CES-D Scale in diverse populations are well established [20,21]. There is internal consistency for the CES-D Scale in both the general population (Cronbach’s alpha = 0.84) and among postpartum women (0.88–0.91) [22]. The Scale allows respondents to indicate the presence of depressive symptoms, such as sadness, crying, hopelessness, or changes in appetite or sleep. The CES-D Scale has a sensitivity of 80–90% in primary care settings with a cutoff of 16 or more. Prior studies have used the CES-D Scale in pregnancy with a moderate sensitivity of 80% [1] and a specificity of 98-99% [1,23]. The CES-D was used in the current study because it was the primary tool used by providers at each of the clinical sites as part of their prenatal assessments. Also, using the CES-D provided an opportunity to compared results in pregnant women with general populations of childbearing women using a similar instrument to that used in primary care practices. Items on the CES-D Scale are rated on a zero-to-three point response scale. A total score is determined by summing the ratings across all 20 items, with possible scores ranging between 0 and 60. The standard threshold of 16 or greater has been used as an indicator of clinically significant elevations in depressive symptoms in community samples as well as in pregnant women [22]. Forty to fifty percent of individuals with scores at or above 16 would be classified as clinically depressed.

#### Social support

Social support was assessed at baseline with using three questions adapted from the Norbeck Social Support (NSSQ) Questionnaire (questions 1,4, and 9) [24]. The NSSQ is an instrument developed for use in pregnancy and allows subjects to list and rate their own social support network by naming persons available for support and then indicated how much support is available from these individuals in daily situations. The reliability and validity of the NSSQ in diverse populations is well established. There is internal consistency in diverse groups of women (Cronbach’s alpha = 0.88-0.91). Participants were asked the following questions: (1) “Do you get emotional support from your spouse/boyfriend/significant other?” (2) “How much of your support is provided by your spouse/boyfriend/significant other?” and (3) “Who provides most of your emotional support?”

#### Clinical

Clinical factors included gestational age, body mass index (BMI) at baseline, prior adverse birth outcomes (preterm birth or spontaneous abortion), past medical conditions and current pregnancy complications. Prior medical conditions were abstracted from electronic medical records at the time of entry into the study and included chronic hypertension, heart disease, diabetes mellitus, sexually transmitted disease, infertility, renal disease, asthma and prior diagnosis of depression. Current pregnancy complications included hypertension, heart disease and asthma, cervical dysplasia requiring colposcopy, pyelonephritis, first or second trimester vaginal bleeding, sexually transmitted disease (gonorrhea, chlamydia, syphilis, hepatitis B) or the diagnosis of depression. Due to small numbers prior medical conditions and current pregnancy-related complicated were re-categorized as composite variables and modeled as dichotomous variables (none versus one or more).

### Statistical analysis

Socio-demographic and clinical factors were compared between black and white women using the χ^2^ statistic for categorical factors (e.g. marital status) and t test for continuous variables (maternal age, gestational age, BMI). Although functional status scores were not normally distributed, the results did not differ with use of nonparametric (Wilcoxon rank-sum test) versus parametric (*t* test) methods. Thus, for ease of interpretation, we present our findings as mean HRQoL scores and 95% confidence intervals. With an alpha of 0.05, there was over 80% power to detect a six point difference or higher in health-related quality of life scores among women in the two racial groups. A difference of six or more points is considered to be an important clinical difference in functioning [25]. In bivariate analysis, we measured the association of demographic and clinical factors, depressive symptoms and social support with maternal HRQoL scores, using analysis of variance. Potential collinearity between socio-demographic variables was examined using a correlation matrix (p = 0.7) and the variance inflation factor.

The presence, magnitude and direction of association of each category of explanatory variables with each domain of health-related quality of life were estimated using multiple linear regression analysis. Each regression coefficient represents, on average, the direction and magnitude of difference in functional status scores between black and white women. Separate linear regression models were developed for each of the eight dimensions of functional status. Variables for the multivariate models were selected on the basis of a priori hypotheses or bivariate associations. In a stepwise fashion, groups of explanatory (predisposing, enabling and clinical) variables were added to the model according to the Institute of Medicine access to care model: [26] first, predisposing variables (age, race, marital status, parity) enabling factors (payment source, education, employment status), third psychosocial factors (depressive symptom level, presence and extent of social support,) and finally clinical factors (gestational age, BMI, history of one or more prior preterm births or spontaneous abortions one or more prior medical conditions, presence of one or more current medical conditions). Multivariate analysis was conducted with and without the participant with a prior history of depression and there was no substantial difference in the adjusted regression coefficients. The Bonferroni correction was used to adjust for multiple testing [27]. The individual contribution of each explanatory factor to the models was assessed by the amount of variation explained (r^2^)^.^ P-values less than 0.05 were considered significant. All analyses were conducted using STATA statistical software (Release 9).

## Results

Of the 221 potentially eligible participants, 195 women agreed to participate (88%). We limited our analysis to 103 black (59% and 72 white women; 20 women had other race/ethnicity. There were no substantial differences in age, parity or number of pregnancy complications in the women excluded from the current analysis. Black women were younger, less educated, and more likely to be single, unemployed, and on Medicaid, compared to their white counterparts (all P-values < 0.001). Twenty-two percent of black women reported depressive symptoms, as measured by the CES-D, compared to 7% of white women (Table 1). Black and white women reported receiving social support from a spouse/significant other, but a statistically significant smaller proportion of Black women reported receiving a great deal of social support (68% versus 88%) compared to white women. Black women had a higher BMI compared to their white counterparts (p = 0.002). Average gestational age at recruitment and enrollment was greater among Black (11±3 weeks) compared to white women (8±4 weeks). There were no differences in parity, prior adverse birth outcomes, or chronic or current medical conditions. Among white women, there was one participant who reported a past history of depression.

**Table 1:** Predisposing, enabling, psychosocial and clinical factors by patient race in the Health Status in Pregnancy (HIP) study (N = 175)

**Factors**	**Participant race**		**P-value**^**1**^
	**Black**	**White**	
	**(N = 103)**	**(N = 72)**	
Predisposing			
Age, yrs, mean ± SD	25 ± 0.6	33 ± 0.6	< 0.001*
Single	73	11	< 0.001*
Parity			0.2
None	2	3	
1 prior birth	27	37	
≥ 2 prior births	71	60	
Education, yrs			< 0. 001*
< 12	26	3	
≥12	74	97	
Enabling			
Unemployed	47	13	< 0.001*
Payment source			< 0.001*
Commercial	65	97	
Medicaid	35	3	
Psychosocial			
Depressive symptoms^2^	22	7	0.02*
Social Support^3^			
Support by significant other	98	99	0.6
Amount of support			0.02*
Little or moderate	22	12	
A great deal	68	88	
Most support other than significant other	56	32	0.03*
Clinical			
Body mass index, mean ± SD	27.8 ± 8	23.2 ± 6.1	0.002*
Gestational age (mean ± SD weeks)	11 ± 3	8 ± 4	< 0.001*
Smoker	10	6	0.7
Prior preterm birth or spontaneous miscarriage	61	46	0.2
≥ 1 Prior medical conditions^4,5^	72	67	0.5
≥ 1 Pregnancy complications^6^	27	24	0.4

Results are reported as percentages unless otherwise indicated. *P-value < 0.05.

^1^P-values are based on the chi square for categorical variables and t-tests for continuous variables.

^2^Depressive symptomatology is based on a Center for Epidemiologic Studies.

Depression (CESD) score of 16 or higher; ^3^ Measures of social support are based on a modified version of the Norbeck Social Support Questionnaire.

^4^Prior medical conditions included chronic hypertension, heart disease, diabetes mellitus, sexually transmitted disease, infertility, renal disease, asthma and prior diagnosis of depression.

^5^Among the 72 white women, one participant reported a prior history of depression.

^6^Pregnancy-related conditions included hypertension, heart disease and asthma, cervical dysplasia requiring colposcopy, pyelonephritis, first or second trimester vaginal bleeding, sexually transmitted disease (gonorrhea, chlamydia, syphilis, hepatitis B).

### Racial differences in HRQoL scores

Black women had statistically significantly lower scores in all four dimensions of physical functioning compared to white women (Table 2). Also, black women had significantly lower scores in Vitality, Social Functioning and Role-Emotional relative to their white counterparts. There were no significant racial differences in scores for Mental Health.

**Table 2:** Health-related quality of life scores in early pregnancy by race, the Health Status in Pregnancy (HIP) study

	**Patient race**		
**HRQoL domains**^**1**^	**Black (n = 103)**	**White (n = 72)**	**P-value**
	**Mean (95% CI)**	**Mean (95% CI)**	
Physical functioning	58 (53–65)	77 (70–84)	< 0.001*
Role-physical	52 (42–63)	78 (66–89)	< 0.001*
Bodily pain	69 (63–74)	83 (77–89)	0.02*
General health	71 (66–76)	83 (79–97)	0.001*
Vitality	47 (42–53)	58 (51–65)	0.01*
Social functioning	72 (66–78)	87 (81–93)	0.002*
Role-emotional	63 (53–74)	89 (80–95)	< 0.001*
Mental health	79 (75–83)	83 (79–86)	0.2

HRQoL = health-related quality of life; 95% CI = 95% confidence interval; * Denotes P-value < 0.05 and therefore, the mean HRQoL score and 95% CI among black women is statistically significantly different from the mean score among white women. P-values are based on a comparison of means using the t-test.

^1^Based on the Medical Outcomes Study (SF-36) Survey.

### Factors associated with physical functioning

In the unadjusted model, black women had statistically significantly lower scores in Physical Functioning, Role-Physical, Bodily Pain and General Health (Table 3). For example, black women had scores that were 14 points lower in Physical Functioning, 8 points lower in Role-Physical, 4 points lower in Bodily Pain and 8 points lower in General Health relative to white women. After the addition of demographic factors, black women still had lower HRQoL scores in Physical Functioning and General Health compared to white women (Table 3). After adjustment for depressive symptoms, social support and BMI, the association of black race with General Health was no longer statistically significant. Further adjustment for prior adverse birth outcomes did not alter the results.

**Table 3:** Association of race with physical functioning, role-physical, bodily pain and general health in early pregnancy: the Health Status in Pregnancy (HIP) study

**Adjustments**	**Black versus white race**			
	**[Beta coefficient**^**1 **^**(95% confidence interval)]**			
	**Physical functioning**	**Role-physical**	**Bodily pain**	**General health**
Model 1: Unadjusted	−14^┼^ (−20, -7)	−8^┼^ (−19, -4)	−14 ^┼^ (−20, -7)	−8 ^┼^ (−12, -4)
Model 2: model 1 + demographic factors^a^	−10^┼^ (−18, -1)	−6 (−21, 9)	−7 (−15, 1)	−5^┼^ (−10, -0.8)
Model 3: model 2 + socioeconomic factors^b^	−10^┼^ (−17, -0.8)	−6 (−21, 8)	−7 (−16, 0.7)	−6^┼^ (−10, -1.0)
Model 4: model 3 + depressive symptoms	−9^┼^ (−17, -1.0)	−6 (−21, 10)	−15^┼^ (−16, -1)	−5^┼^ (−10, -0.6)
Model 5: model 4 + social support^c^	−11^┼^ (−20, -2)	−12^┼^ (−29, 6)	−10^┼^ (−20, -0.3)	−5^┼^ (−10, -0.3)
Model 6: model 5 + pre-pregnancy BMI	−10^┼^ (−19 -0.05)	−13 (−31, 6)	−9 (−19 1.1)	−5 (−10, 0.2)
Model 7: model 6 + prior adverse birth outcomes^d^	−10 (−20, 0.8)	−15 (−31, 3)	−9 (−19, 1)	−5 (−10, 0.8)
Model 8: model 7 + past medical conditions^e^	−11^┼^ (−21, -0.2)	−17 (−36, 1)	−10 (−20,0.5)	−5 (−10, 2)
Model 9: model 8 + current pregnancy Complications^f^	−10 (−20, 0.06)	−18 (−37, 1)	−10 (−20, 0.6)	−5 (−10, 2.0)

BMI = body mass index.

^1^The beta coefficient represents on average the difference in health-related quality of life scores among African-American compared to white women for each functional status domain.

^a^Demographic factors (age, marital status, parity, gestational age).

^b^Socioeconomic factors (education, work status, insurance).

^c^ Social support is based on modified version of the Norbeck Social support Questionnaire.

^d^Adverse birth outcomes (one or more spontaneous abortions or preterm birth).

^e^Past medical conditions include chronic hypertension; heart disease; diabetes mellitus; sexually transmitted disease, infertility; renal disease; prior diagnosis of depression and asthma.

^f^Current pregnancy complications included pregnancy-induced hypertension, heart disease, asthma, cervical dysplasia requiring colposcopy, renal disease or pyelonephritis; first or second trimester vaginal bleeding, sexually transmitted disease (gonorrhea, chlamydia, syphilis, hepatitis B).

^┼^ A confidence interval that excludes 0 indicates statistical significance.

### Factors associated with social functioning

In unadjusted analysis, black race was associated with lower HRQoL scores in Vitality and Social Functioning (Table 4). After adjustment for socio-demographics, black race was still associated with lower HRQoL scores, but the association was no longer statistically significant. Black women on average had lower scores in Role-Emotional and Mental Health compared to white women, but these relationships were not statistically significant.

**Table 4:** Association of race with vitality, social functioning, role-emotional and mental health in early pregnancy: the Health Status in Pregnancy (HIP) study

**Adjustments**	**Black versus white race**			
	**[Beta coefficient**^**1 **^**(95% confidence interval)]**			
	**Vitality**	**Social functioning**	**Role-emotional**	**Mental health**
Model 1: Unadjusted	−4^┼^ (−8, -0.3)	- 7^┼^ (−14, -0.2)	−8 (−18, 3)	−3 (−8, 2)
Model 2: model 1 + demographic factors^a^	−2 (−7, 3)	−5 (−14, 3)	−1 (−13, 14)	−2 (−8, 5)
Model 3: model 2 + socioeconomic factors^b^	−2 (−7, 3)	−6 (−14, 3)	−1 (−15, 13)	−2 (−8, 4)
Model 4: model 3 + depressive symptoms^c^	−2 (−7, 3)	−5 (−13, 4)	3 (−9, 17)	−0.8 (−7, 6)
Model 5: model 4 + social support factors^d^	−4 (−10. 0.2)	−8 (−16, 1)	2 (−11, 15)	−0.2 (−7, -6)
Model 6: model 5 + pre-pregnancy BMI	−4 (−10, 0.5)	−9 (−18, 0.4)	−1 (−15, 12)	−0.7 (−7, 5)
Model 7: model 6 + prior adverse birth outcomes^e^	−4 (−10, 1)	−8 (−18, 1.3)	−2 (−16, 12)	−0.8 (−8, 6)
Model 8: model 7 + chronic medical conditions^f^	−4 (−10, 1)	−8 (−18, 0.8)	−2 (−16, 12)	−0.7 (−8, 7)
Model 9: model 8 + current medical conditions^g^	−4 (−10, 2.0)	−9 (−19, 0.8)	−0.25 (−14, 15)	−0.08 (−7, 6)

BMI = body mass index; ^1^The beta coefficient represents on average the difference in health-related quality of life scores among African-American compared to white women for each functional status domain.

^┼^ A confidence interval that excludes 0 indicates statistical significance.

^a^Demographic factors (age, marital status, parity, gestational age).

^b^Socioeconomic factors (education, work status, payment source).

^c^ Based on Center for Epidemiologic Studies Depression (CES-D) Scale score of 16 or higher.

^d^ Social support is based on modified version of the Norbeck Social support Questionnaire.

^e^Adverse birth outcomes (one or more spontaneous abortions, or preterm birth, or spontaneous abortion).

^f^Past medical conditions include chronic hypertension; heart disease; diabetes mellitus; sexually transmitted disease, infertility; renal disease; prior diagnosis of depression and asthma. ^g^Current pregnancy complications included pregnancy-induced hypertension, heart disease, asthma, cervical dysplasia requiring colposcopy, renal disease or pyelonephritis; first or second trimester vaginal bleeding, sexually transmitted disease (gonorrhea, chlamydia, syphilis, hepatitis B).

## Discussion

While many studies document racial differences in medical outcomes, elucidating or excluding pathways to explain these differences can assist in the development of interventions to eliminate them. This paper summarizes the findings from a cross-sectional study using an established survey validated in pregnant women, [18] to better understand the relation of race with HRQoL during early pregnancy. Racial differences were identified in Physical Functioning, Bodily Pain, General Health, Vitality and Social Functioning. After adjustment for potential confounders, the differences in HRQoL scores were no longer statistically significant. Incremental adjustment (Table 3 and Table 4) indicated that much of the racial variation in physical functioning was due to insurance status and social support. Variation in social functioning was largely explained by the presence of social support.

Recent studies have emphasized the importance of HRQoL within the broader context of maternal health, pregnancy outcomes and neonatal growth and development. If poor functioning in pregnancy is associated with an increased risk of preterm birth [14,28] and higher resource utilization, [29,30] substantive prenatal interventions should be explored. A multidisciplinary approach to improving quality of life includes clinicians, social workers, and community resources, may be needed to promote better functioning during the course of pregnancy. The development of preconception-based interventions that address social support and depression symptoms before conception and extend into the early pregnancy period, may prove effective at increasing early pregnancy functioning.

The findings of this study are largely consistent with other studies in non-pregnant adults that report lower ratings of quality of life among blacks compared to whites [31,32]. In contrast to other studies, however, the presence of medical conditions did not appear to have a substantial effect on HRQoL among women in early pregnancy. There were no statistically significant racial differences in the prevalence of chronic or current medical conditions in our sample of women. Moreover, adjustment for chronic or current conditions did not contribute substantially to the overall variation in physical or social functioning between racial groups. It may be that the effect of pregnancy-specific complications on physical and social functioning is cumulative and increases over the entire course of pregnancy, rather than contributing to a substantive effect in early pregnancy. Alternatively, both black and white women may expect to experience symptoms related to medical conditions or some physical discomfort during pregnancy, with the result that it does not influence their perception of their health-related quality of life.

Social support accounted for a modest amount of the variation in each dimension of physical functioning. Perceived social support has been correlated with depressive symptoms in some studies, particularly among pregnant women [33-35]. It may be that the black women in our study had different perceptions of partner support compared to white women. Social support networks other than spouses or significant others should be considered in future studies of psychosocial factors and their relation to perceptions of quality of life. Because racial differences in patients’ attitudes and preferences for management of psychosocial issues have been reported, [36-38] health care providers caring for expectant mothers should consider patients’ cultural, social and socioeconomic context when negotiating referrals for psychosocial interventions [37,39]. Peer-mentoring among first-time mother has been shown to be effectively in improving infant health [40,41]. Proposed interventions might include similar peer support groups where women can interact with other mothers experiencing depressive symptoms or mothers with a prior history of depression symptoms.

Differences in depressive symptoms between black and white women in our study is similar to other studies among pregnant women [8,42]. Orr and colleagues reported in the rural south that 49% of Black women had CES-D scores of 16 or greater compared with 33% of white women, corresponding to a difference of 16 percentage points [42]. In our sample, there was a 15 percentage point difference between black and white women (22% versus 7%). Findings from the current study confirm that depressive symptoms are prevalent in early pregnancy among a diverse population and suggest the need for effective and efficient screening measures [23].

There are several limitations of this study. First, because the sample included only black and white women from one urban area, the findings may not be generalizable to women of other racial groups or in other geographical regions. However, this survey has been established as a reliable instrument for measuring functional status and has been used in multiple populations in health services research. Depressive symptoms were measured rather than the diagnosis of clinical depression. However, subclinical depression, as a consequence of its high prevalence, is a significant clinical problem as manifested by its effect on health service use and social morbidity among adults in the general population, and our measure is one that has been well validated in numerous populations and settings. Also, there were other potential confounders we were unable to adjust for in the analysis (e.g. domestic violence) that might alter the presence or magnitude of associations between race and HRQoL. Regressions models were adjusted for the presence and level of partner support, but support from other sources than the partner was (e.g. family members, neighbors) was not collected or adjusted for in the multivariate analysis. The linear regression models were also not adjusted for women’s pre-pregnancy lifestyle behaviors or desire for pregnancy on physical and mental functioning during pregnancy. Future studies might assess the influence of pregnancy intent on physical and mental functioning during pregnancy. Rather than adjusting for past and current medical conditions individually in the regression analysis, these conditions were adjusted for as a composite variable (one or more medical conditions versus none). This composite variable is heterogeneous and may have reduced the magnitude of association of race with quality of life, as some conditions may have a greater impact on quality of life than others.

## Conclusions

The findings of this study show that health-related quality of life in early pregnancy deserves further attention. Comprehensive assessment of psychosocial factors during the first prenatal visits can improve functional status in early pregnancy and could affect perceptions of quality of life throughout the course of pregnancy. Training prenatal providers to assess social support and depressive symptoms can serve to improve women’s perceptions of their quality of life. Prenatal providers might collaborate with mental health providers and social work personnel to address physical and social functioning in pregnant women.

## Competing interests

The authors declare that they have no competing interests.

## Authors’ contributions

LL was involved in the conceptual framework of the study. She conducted the statistical analysis and was a primary writer. At the time this study Dr. Liu was a PhD candidate in the Department of Population and Family Health Sciences at Johns Hopkins Bloomberg School of Public Health. WN contributed to the conceptual framework and research hypotheses. She advised Dr. in the conduct of the statistical analysis and made substantive contributions to the writing of the manuscript. Dr. Nicholson was funded, in part, by the American Gynecological and Obstetrical Society and the National Institute for Diabetes and Digestive and Kidney Diseases (5 K23 DK-067944). NRP contributed to interpretation of study results and provided revisions to the text of the manuscript. Dr. Powe was supported by the National Institute for Diabetes and Digestive and Kidney Disease (K24 DK02643). RG served as the lead recruiter and provided leadership in participant retention and data. RS contributed to the interpretation of study results and revisions to the manuscript. All authors read and approved the final manuscript.

## Pre-publication history

The pre-publication history for this paper can be accessed here:

http://www.biomedcentral.com/1471-2393/13/125/prepub

## References

[B1] Chen H Chan YH 3rd Tan KH Lee T Depressive symptomatology in pregnancy - a Singaporean perspective Soc Psychiatry Psychiatr Epidemiol 2004 39 12 975 979 10.1007/s00127-004-0823-8 15583905

[B2] Howell EA Mora PA Horowitz CR Leventhal H Racial and ethnic differences in factors associated with early postpartum depressive symptoms Obstet Gynecol 2005 105 6 1442 1450 10.1097/01.AOG.0000164050.34126.37 15932842 PMC4302723

[B3] Nicholson WK Setse R Hill-Briggs F Cooper LA Strobino D Powe NR Depressive symptoms and health-related quality of life in early pregnancy Obstet Gynecol 2006 107 4 798 806 10.1097/01.AOG.0000204190.96352.05 16582115

[B4] Setse R Grogan R Pham L Cooper LA Strobino D Powe NR Nicholson W Longitudinal study of depressive symptoms and health-related quality of life during pregnancy and after delivery: the Health Status in Pregnancy (HIP) study Matern Child Health J 2009 13 5 577 587 10.1007/s10995-008-0392-7 18931832

[B5] Zayas LH Cunningham M McKee MD Jankowski KR Depression and negative life events among pregnant African-American and Hispanic women Womens Health Issues 2002 12 1 16 22 10.1016/S1049-3867(01)00138-4 11786288

[B6] Emmanuel E St John W Sun J Relationship between social support and quality of life in childbearing women during the perinatal period J Obstet Gynecol Neonatal Nurs. Nov-Dec 2012 41 6 E62 E70 10.1111/j.1552-6909.2012.01400.x 22861382

[B7] Hueston WJ Kasik-Miller S Changes in functional health status during normal pregnancy J Fam Pract 1998 47 3 209 212 9752373

[B8] Haas JS Jackson RA Fuentes-Afflick E Stewart AL Dean ML Brawarsky P Escobar GJ Changes in the health status of women during and after pregnancy J Gen Intern Med 2005 20 1 45 51 10.1111/j.1525-1497.2004.40097.x 15693927 PMC1490030

[B9] McKee MD Cunningham M Jankowski KR Zayas L Health-related functional status in pregnancy: relationship to depression and social support in a multi-ethnic population Obstet Gynecol 2001 97 6 988 993 10.1016/S0029-7844(01)01377-1 11384708

[B10] Haas JS Meneses V McCormick MC Outcomes and health status of socially disadvantaged women during pregnancy J Womens Health Gend Based Med 1999 8 4 547 553 10.1089/jwh.1.1999.8.547 10839710

[B11] Darcy JM Grzywacz JG Stephens RL Leng I Clinch CR Arcury TA Maternal depressive symptomatology: 16-month follow-up of infant and maternal health-related quality of life J Am Board Fam Med 2011 24 3 249 257 10.3122/jabfm.2011.03.100201 21551396 PMC3114440

[B12] Negron R Martin A Almog M Balbierz A Howell EA Social support during the postpartum period: mothers’ views on needs, expectations, and mobilization of support Matern Child Health J 2012 17 4 616 623 22581378 10.1007/s10995-012-1037-4 PMC3518627

[B13] Haas JS Fuentes-Afflick E Stewart AL Jackson RA Dean ML Brawarsky P Escobar GJ Prepregnancy health status and the risk of preterm delivery Arch Pediatr Adolesc Med 2005 159 1 58 63 10.1001/archpedi.159.1.58 15630059

[B14] Goldenberg RL Culhane JF Prepregnancy health status and the risk of preterm delivery Arch Pediatr Adolesc Med 2005 159 1 89 90 10.1001/archpedi.159.1.89 15630064

[B15] Muzik M Borovska S Perinatal depression: implications for child mental health Ment Health Fam Med 2010 7 4 239 247 22477948 PMC3083253

[B16] Minkovitz CS Strobino D Scharfstein D Hou W Miller T Mistry KB Swartz K Maternal depressive symptoms and children’s receipt of health care in the first 3 years of life Pediatrics 2005 115 2 306 314 10.1542/peds.2004-0341 15687437

[B17] Ware J SF-36 Health Survey. Manual and interpretation guide 1993 The Health Institute, New England Medical Center

[B18] Jomeen J Martin CR The factor structure of the SF-36 in early pregnancy J Psychosom Res 2005 59 3 131 138 10.1016/j.jpsychores.2005.02.018 16198185

[B19] Stewart AL Greenfield S Hays RD Wells K Rogers WH Berry SD McGlynn EA Ware JE Jr Functional status and well-being of patients with chronic conditions. Results from the Medical Outcomes Study JAMA 1989 262 7 907 913 10.1001/jama.1989.03430070055030 2754790

[B20] Radloff L A self-report depression scale for research in the general population Appl Psychol Meas 1977 1 385 401 10.1177/014662167700100306

[B21] Roberts RE Reliability of the CES-D Scale in different ethnic contexts Psychiatry Res 1980 2 2 125 134 10.1016/0165-1781(80)90069-4 6932058

[B22] Marcus SM Flynn HA Blow FC Barry KL Depressive symptoms among pregnant women screened in obstetrics settings J Womens Health (Larchmt) 2003 12 4 373 380 10.1089/154099903765448880 12804344

[B23] Gaynes BN Gavin N Meltzer-Brody S Lohr KN Swinson T Gartlehner G Brody S Miller WC Perinatal depression: prevalence, screening accuracy, and screening outcomes Evid Rep Technol Assess (Summ) 2005 119 1 8 15760246 10.1037/e439372005-001 PMC4780910

[B24] Norbeck JS Lindsey AM Carrieri VL Further development of the norbeck social support questionnaire: normative data and validity testing Nurs Res 1983 32 1 4 9 6549842

[B25] McHorney CA Ware JE Jr Lu JF The SCD MOS 36-Item short-form health survey (SF-36): III. Tests of data quality, scaling assumptions, and reliability across diverse patient groups Med Care 1994 32 1 40 66 10.1097/00005650-199401000-00004 8277801

[B26] Institute of Medicine Access to Care in America: A Model for Providing Care 1993 Washington, DC: Washington Academy Press

[B27] Hochberg YBY More powerful procedures for multiple significance testing Stat Med 1990 9 7 811 818 10.1002/sim.4780090710 2218183

[B28] Neggers Y Goldenberg R Cliver S Hauth J The relationship between psychosocial profile, health practices, and pregnancy outcomes Acta Obstet Gynecol Scand 2006 85 3 277 285 10.1080/00016340600566121 16553174

[B29] Druss BG Rosenheck RA Sledge WH Health and disability costs of depressive illness in a major U.S. corporation Am J Psychiatry 2000 157 8 1274 1278 10.1176/appi.ajp.157.8.1274 10910790

[B30] Egede LE Major depression in individuals with chronic medical disorders: prevalence, correlates and association with health resource utilization, lost productivity and functional disability Gen Hosp Psychiatry 2007 29 5 409 416 10.1016/j.genhosppsych.2007.06.002 17888807

[B31] Collins KSTK Hughes DL Quality of health care for African-Americans: Findings from The Commonwealth Fund 2001 Health Care Quality Survey 2002 New York: NY: The Commonwealth Fund Press *Publication* #*524*. New York: NY2002

[B32] Hill-Briggs F Gary TL Baptiste-Roberts K Brancati FL Thirty-six-item short-form outcomes following a randomized controlled trial in type 2 diabetes Diabetes Care 2005 28 2 443 444 10.2337/diacare.28.2.443 15677813

[B33] Elsenbruch S Benson S Rucke M Rose M Dudenhausen J Pincus-Knackstedt MK Klapp BF Arck PC Social support during pregnancy: effects on maternal depressive symptoms, smoking and pregnancy outcome Hum Reprod 2007 22 3 869 877 17110400 10.1093/humrep/del432

[B34] Da Costa D Brender W Larouche J A prospective study of the impact of psychosocial and lifestyle variables on pregnancy complications J Psychosom Obstet Gynaecol 1998 19 1 28 37 10.3109/01674829809044218 9575466

[B35] Da Costa D Dritsa M Larouche J Brender W Psychosocial predictors of labor/delivery complications and infant birth weight: a prospective multivariate study J Psychosom Obstet Gynaecol 2000 21 3 137 148 10.3109/01674820009075621 11076335

[B36] Dwight-Johnson M Sherbourne CD Liao D Wells KB Treatment preferences among depressed primary care patients J Gen Intern Med 2000 15 8 527 534 10.1046/j.1525-1497.2000.08035.x 10940143 PMC1495573

[B37] Givens JL Houston TK Van Voorhees BW Ford DE Cooper LA Ethnicity and preferences for depression treatment Gen Hosp Psychiatry 2007 29 3 182 191 10.1016/j.genhosppsych.2006.11.002 17484934

[B38] Cooper LA Gonzales JJ Gallo JJ Rost KM Meredith LS Rubenstein LV Wang NY Ford DE The acceptability of treatment for depression among African-American, Hispanic, and white primary care patients Med Care 2003 41 4 479 489 12665712 10.1097/01.MLR.0000053228.58042.E4

[B39] Huang ZJ Wong FY Ronzio CR Yu SM Depressive symptomatology and mental health help-seeking patterns of U.S.- and foreign-born mothers Matern Child Health J 2007 11 3 257 267 10.1007/s10995-006-0168-x 17171544

[B40] Murphy CA Cupples ME Percy A Halliday HL Stewart MC Peer-mentoring for first-time mothers from areas of socio-economic disadvantage: a qualitative study within a randomised controlled trial BMC Health Serv Res 2008 8 46 10.1186/1472-6963-8-46 18304334 PMC2291460

[B41] Cupples ME Stewart MC Percy A Hepper P Murphy C Halliday HL A RCT of peer-mentoring for first-time mothers in socially disadvantaged areas (the MOMENTS Study) Arch Dis Child 2011 96 3 252 258 10.1136/adc.2009.167387 20522466

[B42] Orr ST Blazer DG James SA Racial disparities in elevated prenatal depressive symptoms among black and white women in eastern north Carolina Ann Epidemiol 2006 16 6 463 468 10.1016/j.annepidem.2005.08.004 16257228

